# Promiscuous Recognition of a *Trypanosoma cruzi* CD8^+^ T Cell Epitope among HLA-A2, HLA-A24 and HLA-A1 Supertypes in Chagasic Patients

**DOI:** 10.1371/journal.pone.0150996

**Published:** 2016-03-14

**Authors:** Paola Lasso, Lina Beltrán, Fanny Guzmán, Fernando Rosas, M. Carmen Thomas, Manuel Carlos López, John Mario González, Adriana Cuéllar, Concepción J. Puerta

**Affiliations:** 1 Laboratorio de Parasitología Molecular, Pontificia Universidad Javeriana, Bogotá, Colombia; 2 Grupo de Inmunobiología y Biología Celular, Pontificia Universidad Javeriana, Bogotá, Colombia; 3 Instituto de Parasitología y Biomedicina López-Neyra, CSIC, PTS-Granada, Granada, España; 4 Núcleo de Biotecnología Curauma (NBC), Pontificia Universidad Católica de Valparaíso, Valparaíso, Chile; 5 Instituto de Arritmias Joseph Brugada, Fundación Clínica Abood Shaio, Bogotá, Colombia; 6 Grupo de Ciencias Básicas Médicas, Facultad de Medicina, Universidad de los Andes, Bogotá, Colombia; Hospital Israelita Albert Einstein, BRAZIL

## Abstract

**Background:**

TcTLE is a nonamer peptide from *Trypanosoma cruzi* KMP-11 protein that is conserved among different parasite strains and that is presented by different HLA-A molecules from the A2 supertype. Because peptides presented by several major histocompatibility complex (MHC) supertypes are potential targets for immunotherapy, the aim of this study was to determine whether MHC molecules other than the A2 supertype present the TcTLE peptide.

**Methodology/Principal Findings:**

From 36 HLA-A2-negative chagasic patients, the HLA-A genotypes of twenty-eight patients with CD8^+^ T cells that recognized the TcTLE peptide using tetramer (twenty) or functional (eight) assays, were determined. SSP-PCR was used to identify the A locus and the allelic variants. Flow cytometry was used to analyze the frequency of TcTLE-specific CD8^+^ T cells, and their functional activity (IFN-γ, TNFα, IL-2, perforin, granzyme and CD107a/b production) was induced by exposure to the TcTLE peptide. All patients tested had TcTLE-specific CD8^+^ T cells with frequencies ranging from 0.07–0.37%. Interestingly, seven of the twenty-eight patients had HLA-A homozygous alleles: A*24 (5 patients), A*23 (1 patient) and A*01 (1 patient), which belong to the A24 and A1 supertypes. In the remaining 21 patients with HLA-A heterozygous alleles, the most prominent alleles were A24 and A68. The most common allele sub-type was A*2402 (sixteen patients), which belongs to the A24 supertype, followed by A*6802 (six patients) from the A2 supertype. Additionally, the A*3002/A*3201 alleles from the A1 supertype were detected in one patient. All patients presented CD8^+^ T cells producing at least one cytokine after TcTLE peptide stimulation.

**Conclusion/Significance:**

These results show that TcTLE is a promiscuous peptide that is presented by the A24 and A1 supertypes, in addition to the A2 supertype, suggesting its potential as a target for immunotherapy.

## Introduction

Chagas disease is a neglected tropical disease that is caused by the flagellated protozoan parasite *Trypanosoma cruzi* and that is primarily transmitted by blood-sucking triatomines [1]. Approximately 10 million people are infected worldwide, with approximately 40,000 new cases occurring every year, and the estimated burden of this disease in terms of disability-adjusted life years (DALYs) is 586,000 [1,2]. After the acute phase, 70–80% of infected people remain asymptomatic throughout their lives, whereas the other 20–30% develop chronic cardiac or digestive diseases, which can lead to death [1,2]. Unfortunately, beyond vector control measures, no prophylactic measures currently exist, and the available treatments are not only toxic but also have not been proven to be effective in cases of chronic chagasic patients [1,2].

CD8^+^ T cells are a critical component of the protective immune response against the intracellular parasite *T*. *cruzi* [3]. The depletion of CD8^+^ T cells in animal models of *T*. *cruzi* acute infection demonstrated the role of these cells in infection control. For example, in CD8^+^ T cell-depleted mice, the disease develop faster, and these animals have higher parasitemia levels than wild-type infected mice [4–8]. CD8^+^ T cell activation occurs when cells specifically recognize short peptides (8 to 10 amino acids in length), which are usually derived from intracellular proteins and which are presented in the context of the major histocompatibility complex class I (MHC I) molecules [9–11]. Whereas peptide binding to MHC I molecules occurs through an interaction between the side chains of the peptide amino acids and the binding pockets of the MHC I molecule, the peptide-binding affinity is usually determined by an interaction between the amino acids located on the N- and C-termini of the peptide with the B and F pockets of the class I molecules [12,13]. Class I molecules are grouped into diverse clusters or supertypes that share similarities in their peptide-binding pockets. Thus, HLA supertypes are defined as a set of alleles with potentially similar binding pockets that allow the binding of related peptides [13,14]. At least nine MHC class I supertypes have been described: A1, A2, A3, A24, B7, B27, B44, B58 and B62 [13–15].

The frequencies of HLA alleles vary greatly among different populations; thus, obtaining knowledge regarding these alleles is a critical step in the development of potential therapeutic or prophylactic immune strategies that require population-wide coverage. In this context, promiscuous peptides, which are defined as peptides that can be presented by different HLA molecules belonging to the same MHC [16] or even different MHC supertypes, are attractive targets for immunotherapy [17–20].

Our group recently reported that the TcTLE peptide (previously named K1 peptide) from the KMP-11 protein of *T*. *cruzi* is a HLA-A*0201-restricted peptide [21]. This peptide is recognized during the natural course of the disease and is presented by HLA-A molecules of the A2 supertype, such as HLA-A*0205, HLA-A*0222, HLA-A*0226, HLA-A*0259 and HLA-A*0287 [22].

In the present study, the HLA-A2-restricted TcTLE peptide was observed to be presented by other HLA-A supertypes and able to induce functional activity in CD8^+^ T cells from these chagasic patients. Indeed, the finding that A24, A23 and A01 homozygous patients have TcTLE-specific CD8^+^ T cells demonstrated that several MHC supertypes present this peptide. TcTLE peptide-specific CD8^+^ T cells from non-HLA-A2 chagasic patients were also shown to be able to produce gamma interferon (IFN-γ), tumoral necrosis factor (TNFα), interleukin-2 (IL-2), perforin, and granzyme B and to express the proteins CD107a/b in response to TcTLE peptide stimulation.

## Material and Methods

### Human study population

Thirty-six chagasic patients from endemic areas of Colombia, including 13 males and 23 females with ages ranging from 19–76 years, were included in the study. All the subjects were recruited and clinically evaluated at the Hospital Universitario San Ignacio, Fundación Abood Clínica Shaio or Instituto Nacional de Salud in Bogotá, Colombia. All subjects were anti-*T*. *cruzi* antibody-positive according to both indirect immunofluorescence immunoassay (IFI) and enzyme-linked immunosorbent assay (ELISA) [23]. All chagasic patients were classified according to the Kuschnir grading system [24]. Fifteen patients were classified as non-cardiac chagasic patients or G0 stage (normal electrocardiogram (ECG) results), and twenty-one were classified as cardiac chronic chagasic patients (CCC) with different degrees of disease severity as follows: seven G1 with abnormal ECG results, seven G2 with abnormal ECG results and cardiac enlargement and seven G3 with abnormal ECG results, cardiac enlargement and clinical signs of heart failure. The group of healthy donors (HDs) consisted of fourteen seronegative individuals with similar ages to the chagasic donors who have always resided in non-endemic areas and who exhibited normal ECG results and clinical examination. All donors were volunteers, and they signed informed consent forms before being included in the study.

Approximately 20 mL of blood was collected from each individual by venipuncture as follows: 10 mL was collected in heparinized tubes for isolating peripheral blood mononuclear cells (PBMCs) by Ficoll-Paque PLUS density gradient centrifugation (GE Healthcare Bioscience, Uppsala, Sweden), 4 mL was collected in EDTA tubes for DNA extraction and 6 mL was collected for serological tests (Vacutainer, Becton-Dickinson, San José, CA, USA).

### Ethics statement

This work was approved by the Research and Ethics Committees of the Facultad de Ciencias from the Pontificia Universidad Javeriana (6^th^ April 2011 and 17^th^ June 2009 CIEs). All adult subjects who participated in the study read, accepted and signed the informed consent form.

### HLA-A2 typing by flow cytometry

HLA-A2 typing was performed as previously described [22]. Briefly, 100 μL of peripheral blood was incubated with 0.25 μg of anti-HLA-A2 FITC antibody (clone BB7.2; BD Pharmingen, San Diego, CA, USA) for 20 minutes at 4°C [25]. After adding 500 μL of 1X lysis buffer (BD Bioscience, San José, CA, USA), the cells were incubated for 15 minutes at room temperature. Finally, the cells were washed with 1X phosphate-buffered saline (PBS; Eurobio, Les Ulis, France), and data were collected using a FACSAria II flow cytometer (BD Immunocytometry Systems, San José, CA, USA). The data were analyzed with FlowJo 9.3.2 software (Tree Star, Inc., Ashland, OR, USA).

### HLA typing by SSP Unitray

HLA-A locus evaluation was performed using a commercial PCR kit based on sequence-specific primers (SSP-PCR) according to the manufacturer’s instructions (Biotest, Landsteinerstr, Dreieich, Germany). Genomic DNA was extracted from each patient’s peripheral blood using a GFX genomic blood DNA purification kit (Amersham Biosciences, Piscataway, NJ, USA). PCRs were conducted in a Stomacher 3500 Thermal Cycler PTC-100 (MJ Research, Inc., Watertown, MA, USA). The amplified products were resolved via 2% agarose gel electrophoresis and visualized following ethidium bromide staining. The amplified fragments were analyzed using Unimatch^TM^ 4.01 software (Invitrogen, Brown Deer, WI, USA). Once the alleles present in each individual were determined, medium-high-resolution typing was performed using A*01, A*03, A*11, A19, A*23, A*24 or A*68 SSP Unitray kits (Dynal Invitrogen Corporation, Brown Deer, WI, USA) according to the manufacturer’s protocols.

### Tetramer staining

**Fig 1:**
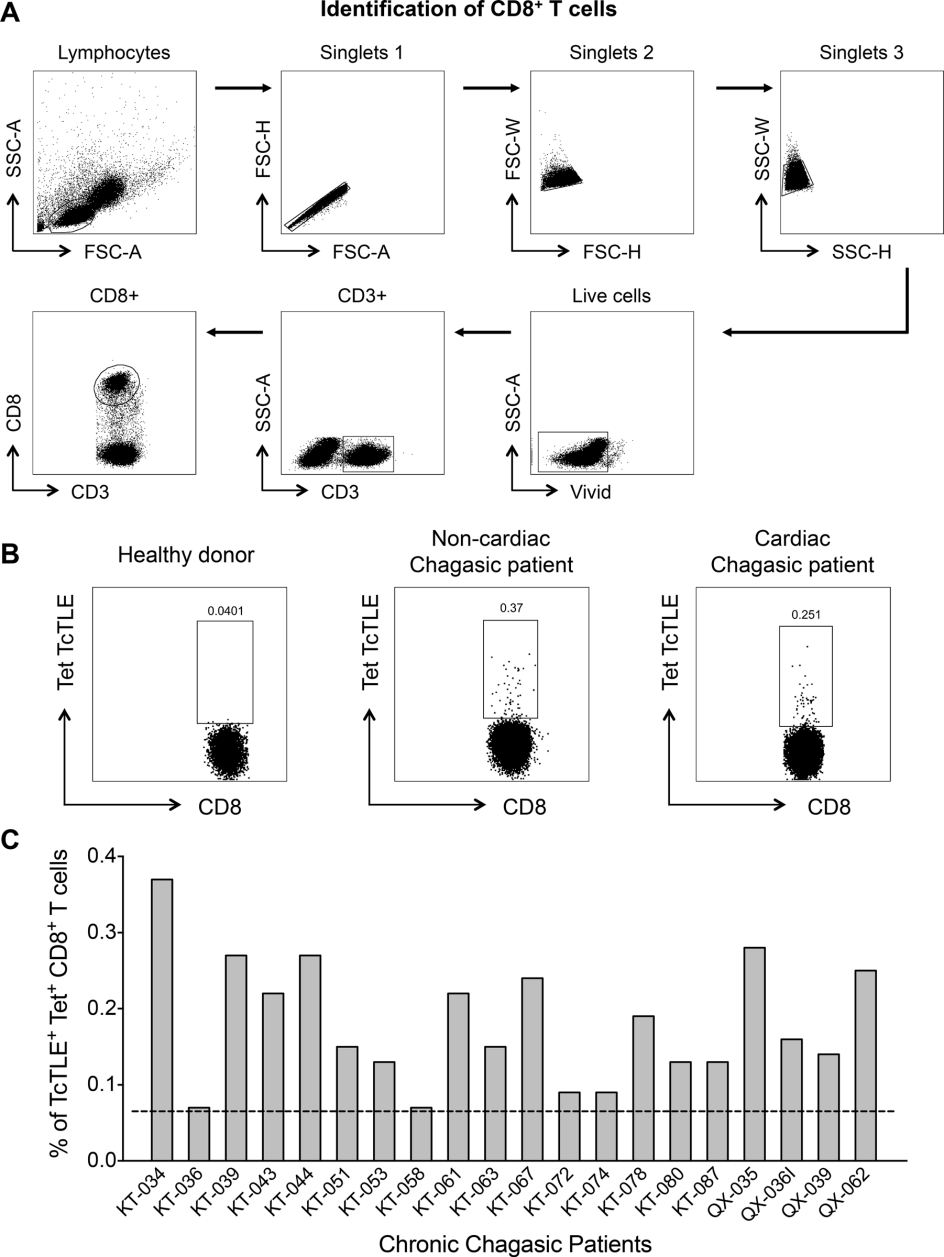
Flow cytometry gating strategy and frequency of TcTLE-specific CD8^+^ T cells. Dot plots representative of the analysis of an HLA-A2-negative chagasic patient. (A) Lymphocytes were selected, and cell doublets were excluded from the analysis for the identification of CD8^+^ T cells based on the forward scatter area (FSC-A) and forward scatter height (FSC-H) (singlet 1), forward scatter width (FSC-W) and forward scatter height (FSC-H) (singlet 2), and side scatter width (SSC-W) and SSC-H characteristics (singlet 3). Finally, live CD3^+^ CD8^+^ cells were selected. (B) Selection of TcTLE peptide-specific CD8^+^ T cells by soluble tetramer assay. (C) Frequency of TcTLE peptide-specific CD8^+^ T cells from 20 HLA-A2-negative chronic chagasic patients. The cut-off point, which was fixed at 0.063, is indicated by the dotted line.

HLA-A2 PE-labeled tetramers loaded with TcTLE peptide (TLEEFSAKL), derived from *T*. *cruzi* KMP-11 protein [22], and also HLA-A2 PE-labeled tetramers with a modified Flu-MP peptide (Flu-MP*; GILGFVTTL) derived from the influenza virus matrix protein [22,26], and a modified CMV pp65 peptide (CMV*; DLSPMVATV), employed as controls, were produced by the National Institute of Health (NIH) Tetramer Facility (Atlanta, GA, USA). In total, 1 × 10^6^ PBMCs per tube were stained with 0.5 μg/mL tetramers and anti-CD3-PerCP (clone SK7) and anti-CD8-FITC (clone SK1) conjugates (BD Biosciences, San José, CA, USA) for 20 minutes in the dark at room temperature. After the cells were washed with staining buffer (1% fetal bovine serum in 1X PBS), they were resuspended in 500 μL of 1X PBS. At least 50,000 events gated for CD3^+^ CD8^+^ T cells were acquired and analyzed using a FACSAria II flow cytometer (BD Immunocytometry Systems) and FlowJo 9.3.2 software (Tree Star, Inc.). The gating strategy is shown in Fig 1A and 1B. The cut-off point for HLA-A2/TcTLE tetramer CD8^+^ T cells was established at 0.063% after determining the average background value of K1-specific CD8^+^ T cells from HD (0.027%) plus three standard deviations (0.012%). A cut-off point for HLA-A2/Flu-MP* and HLA-A2/CMV* tetramer CD8^+^ T cells was fixed at 0.1% [26].

### CD8^+^ T cell cytokine production and degranulation assays by flow cytometry

To assess T cell function, 1 × 10^6^ PBMCs were cultured with the TcTLE peptide (10 μg/mL) for 6 hours at 37°C and 5% CO_2_ in the presence of CD28 (1 μg/mL) and CD49d (1 μg/mL). After the cells were incubated for one hour, brefeldin A (1 μg/mL) and monensin (1 μg/mL) (BD Pharmingen) were added to the cultures. PBMCs were labeled with Live/Dead Fixable Aqua for 20 minutes in the dark at room temperature. Then, the cells were stained with anti-CD3 Pacific Blue (clone UCHT1) and anti-CD8 APC-H7 (clone SK1) monoclonal antibodies (BD Biosciences). The cells were permeabilized with Cytofix/Cytoperm (BD Pharmingen) and stained with anti-IL-2 PerCP-Cy5.5 (clone MQ1-17H12; BD Biosciences), anti-IFN-γ Alexa Fluor 700 (clone B27; BD Biosciences, San Jose, CA, USA), anti-TNFα APC (clone 6401.1111; BD Biosciences), anti-Perforin PE (clone B-D48; Abcam, Cambridge, MA, USA) and anti-Granzyme B Alexa Fluor 647 (clone GB11) conjugates (BD Biosciences) for 30 minutes at 4°C. To evaluate potential cytotoxic activity, anti-CD107a (clone H4A3) and CD107b FITC (clone H4B4) conjugates (BD Biosciences) were added to the PBMCs before stimulation. In each experiment, non-stimulated cells were used as a negative control, and staphylococcal enterotoxin B (SEB) (1.5 μg/mL) (Sigma-Aldrich, Saint Louis, MO, USA) was used as a positive control (S1 and S2 Tables). At least 50,000 events gated on live CD3^+^ CD8^+^ T cells were acquired using a FACSAria II flow cytometer (BD Immunocytometry Systems) and analyzed with FlowJo 9.3.2 software (Tree Star, Inc.). A positive cytokine response was defined by subtracting the background of cytokine production (cells cultured without antigen) and obtaining a value exceeding 0.05%; this frequency corresponds to the average frequency of CD8^+^ T cells producing cytokines from HDs cultured with the TcTLE peptide plus 3 standard deviations. Multifunctional analysis was performed using the Boolean gating strategy and visually represented with Pestle version 1.7 and SPICE software version 5.3 (provided by the NIH, Bethesda, MD, USA) [27].

### Statistical analysis

Differences between two groups were assessed using the Mann-Whitney test. Tests were two-tailed, and differences were considered statistically significant when *p* < 0.05. GraphPad Prism version 6.0 for Mac OS X (GraphPad Software, San Diego, CA, USA) statistical software was used for statistical analyses.

## Results

### TcTLE peptide: An epitope presented by several HLA-A supertypes

Previous studies of the KMP-11-derived TcTLE peptide revealed that it is efficiently processed, presented and recognized by CD8^+^ T cells in the context of the HLA-A*0201 molecule during the natural course of Chagas disease [22,28]. In addition, by applying a tetramer staining approach, the TcTLE peptide was observed to also be presented by other HLA-A2 alleles because it was recognized by CD8^+^ T cells from infected patients in the context of the HLA-A*0205, HLA-A*0222, HLA-A*0226, HLA-A*0259 and HLA-A*0287 alleles [22]. Unexpectedly, when examining the frequency and functionality of TcTLE peptide-specific CD8^+^ T cells in a broader number of patients, twenty-eight of the thirty-six analyzed HLA-A2-negative chagasic patients were found to have CD8^+^ T cells that recognized the TcTLE peptide. Tetramer staining revealed that twenty of these patients had CD8^+^ T cells that were able to bind to the HLA-A2/K1-peptide tetramer at percentages ranging from 0.07–0.37% (median = 0.16%) (Fig 1C and Table 1). The CD8^+^ T cells of the remaining eight patients were evaluated according to cytokine production and cytotoxic activity and were found to recognize the TcTLE peptide and to respond by producing cytokines or by exhibiting cytotoxic activity (see below). In order to evaluate the frequency of specific-CD8^+^ T cells of the irrelevant peptides, used as controls, the frequency of Flu-MP* and CMV* specific-CD8^+^ T cells was determined in the same assay conditions and with cells from the same patients. Indeed, it was found that although 11 out of 28 chagasic patients presented frequencies to both, TcTLE and Flu-MP* peptides, 8 out of 28 only presented frequencies to TcTLE peptide and one patient (KT-059) only presented frequencies to Flu-MP*. Additionally, the frequency of CD8^+^ T cells specific to CMV* was determined in the QX-036 and QX-039 patients. The results showed that while the QX-036 patient had frequencies of CD8^+^ T cells specific to TcTLE and CMV* peptides, the QX-039 patient only had frequencies of TcTLE peptide-specific CD8^+^ T cells. Together, these results demonstrate the specificity of the response.

**Table 1:** Characteristics of HLA-A2^-^ individuals, the frequency of CD8^+^ T cells specific for the TcTLE peptide, and low-resolution typing of HLA-A alleles.

Patient code	Clinical status	Birthplace	Gender	Age	Tetramer-specific CD8^+^ T cells (%)			HLA-A alleles
					TcTLE	Flu-MP*	CMV*	
KT-035	HD	Bogotá—Cundinamarca	F	37	0.03	0.03	ND	A23/A24
KT-094	HD	Bogotá—Cundinamarca	M	67	0.03	0.02	ND	A24/A68
KT-096	HD	Roldanillo—Valle	F	61	0.02	0	ND	A01/A24
KT-097	HD	Barranquilla—Atlántico	F	19	0.04	0.03	ND	A24/A24
KT-098	HD	Bogotá—Cundinamarca	F	58	0.01	0	ND	A30/A33
KT-099	HD	Bogotá—Cundinamarca	M	44	0.01	0.04	ND	A03/A68
KT-100	HD	Bogotá—Cundinamarca	F	39	0.02	0.05	ND	A24/A32
KT-101	HD	Bogotá—Cundinamarca	F	65	0.04	0.01	ND	A24/A30
CS-005	HD	Pasto—Nariño	F	26	0.04	ND	ND	A29/A31
CS-007	HD	Bogotá—Cundinamarca	F	47	ND	ND	ND	A24/A24
CS-016	HD	Bogotá—Cundinamarca	F	41	ND	ND	ND	A24/A68
CS-017	HD	Bogotá—Cundinamarca	M	44	ND	ND	ND	A11/A33
CS-018	HD	Bogotá—Cundinamarca	F	44	ND	ND	ND	A30/A33
CS-040	HD	Medellín—Antioquia	M	58	ND	ND	ND	A03/A30
KT-034	G0	Moniquirá—Boyacá	M	37	**0.37**	**0.29**	ND	A23/ A68
QX-035	G0	Sucre—Santander	F	39	**0.28**	0.09	ND	A24/ A30
QX-036	G0	Gachalá—Cundinamarca	F	44	**0.16**	0.09	**0.26**	A24/ A68
QX-039	G0	Apulo—Cundinamarca^a^	F	54	**0.14**	0.08	0.03	A24/ A29
KT-044	G0	Bogotá—Cundinamarca	M	19	**0.27**	**0.25**	ND	A24/A24
KT-062	G0	Paéz—Boyacá	F	28	0.04	0.05	ND	A11/A11
KT 063	G0	Somondoco—Boyacá	M	57	**0.15**	0.06	ND	A11/ A68
KT-069	G0	Capitancio—Santander	M	45	0.02	0.05	ND	A24/A31
KT-073	G0	Miraflores—Boyacá	M	42	0.03	0.04	ND	A24/A30
KT-081	G0	Paéz—Boyacá	F	56	0.04	0.09	ND	A24/A68
KT 087	G0	Bolivar—Santander	F	71	**0.13**	0.08	ND	A24/ A68
KT 088	G0	Miraflores—Boyacá	M	60	**0.13**	0.09	ND	A24/A24
KT 043	G1	Soata—Boyacá	M	61	**0.22**	**0.33**	ND	A23/ A68
KT-050	G1	Miraflores—Boyacá	F	45	0.04	0.05	ND	A24/A31
KT 067	G1	Ibagué—Tolima	M	41	**0.24**	**0.22**	ND	A01/ A31
KT-068	G1	Gallegos—Santander	F	59	0.05	0.04	ND	A31/A68
KT-071	G1	Rondón—Boyacá	M	38	0.03	0.05	ND	A24/A32
KT 072	G1	Yopal—Casanare	F	52	**0.09**	**0.18**	ND	A23/A23
KT 078	G1	El Engaño—Cundinamarca	F	60	**0.19**	**0.18**	ND	A01/ A29
KT-039	G2	Soatá—Boyacá	F	67	**0.27**	**0.23**	ND	A24/A24
KT-040	G2	Sutatenza—Boyacá	F	76	**0.07**	0.04	ND	A30/ A32
KT-053	G2	Pajarito—Boyacá^a^	F	44	**0.13**	**0.12**	ND	A24/ A68
KT-059	G2	San José de Pare—Boyacá	F	49	0.06	**0.11**	ND	A03/A32
KT-061	G2	Berbeo—Boyacá	M	65	**0.22**	**0.15**	ND	A24/ A31
KT-074	G2	San Joaquín—Santander	M	46	**0.09**	0.09	ND	A11/ A24
KT-051	G3	Guamo—Tolima	F	58	**0.15**	**0.26**	ND	A11/ A23
KT-058	G3	Armero—Tolima	F	39	**0.07**	0.08	ND	A24/ A68
QX-062	G3	Zetaquirá—Boyacá	F	70	**0.25**	**0.22**	ND	A24/ A29
QX-018	G0	Tablón—Togui-Boyacá^a^	F	35	ND ^b^	ND	ND	A24/A24
QX-045	G0	Santander—Belleza	F	32	ND ^b^	ND	ND	A01/A01
QX-050	G0	Chima—Santander	F	39	ND ^b^	ND	ND	A03/A30
QX-052	G2	Chitaraque—Boyacá	F	65	ND ^b^	ND	ND	A29/A31
QX-002	G3	Sabana larga—Casanare	M	65	ND ^b^	ND	ND	A24/A31
QX-031	G3	Soatá—Boyacá	F	55	ND ^b^	ND	ND	A24/A24
QX-051	G3	Chitaraque—Boyacá	F	55	ND ^b^	ND	ND	A11/A24
QX-054	G3	Fómeque—Cundinamarca	M	57	ND ^b^	ND	ND	A24/A68

* Indicates that peptides have a modification compared to original peptide

^a^ Indicates people who continued living in endemic areas. HDs: healthy donors; ND: not determined.

^b^ Indicates patients who have TcTLE-specific CD8^+^ T cells with cytokine production after TcTLE peptide stimulation.

Positive frequencies of specific CD8^+^ T cells are indicated in bold.

**Fig 2:**
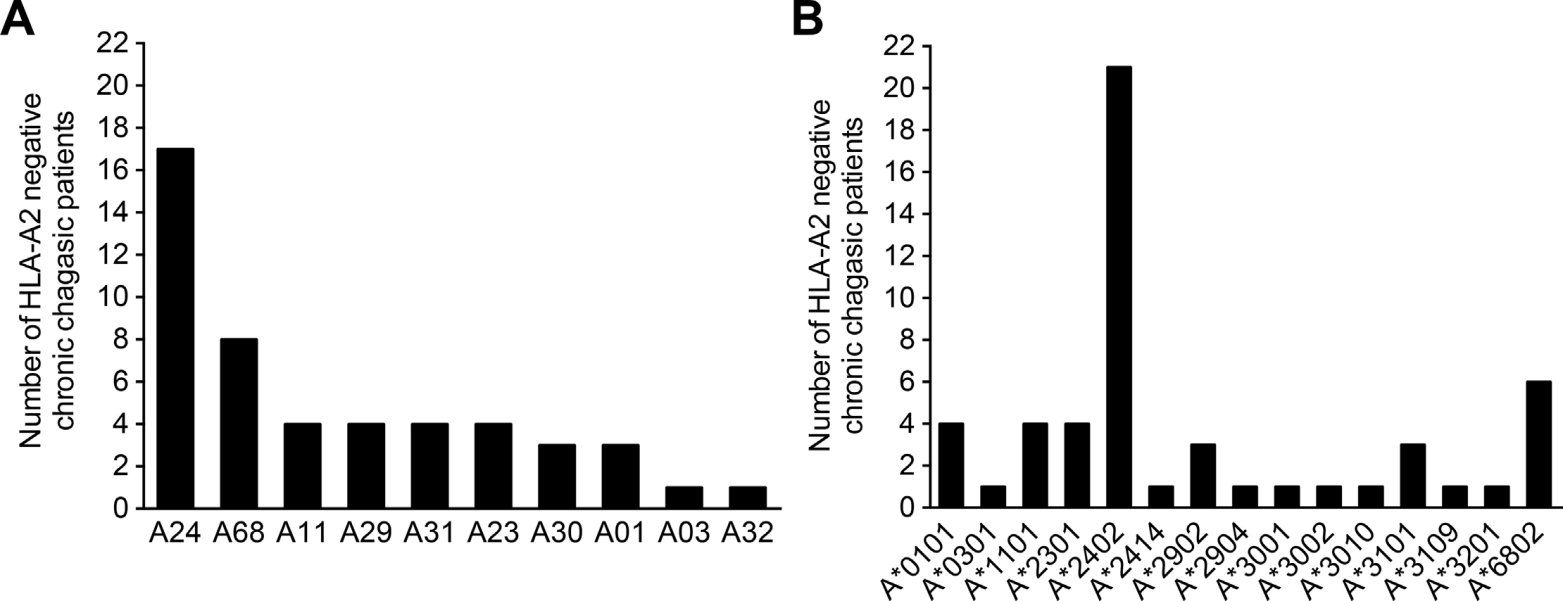
HLA typing of chronic chagasic patients with TcTLE-specific CD8^+^ T cells. HLA-A types (A) and sub-types (B) of chronic chagasic patients with TcTLE peptide-specific CD8^+^ T cells. The figure shows the number of patients with each HLA-A type and sub-type.

Based on these results, the HLA-A genotype of these twenty-eight patients (Table 1) was confirmed by HLA-A locus PCR-SSP molecular typing. Seven of the twenty-eight (25%) tetramer- or cytokine/cytotoxic-positive chagasic patients had HLA-A homozygous alleles for A*24 (five patients), A*23 (one patient), and A*01 (one patient) (Table 1). Among the twenty-one HLA-A heterozygous patients, the most common alleles were A*24 (57.1%) and A*68 (38.0%), followed by A*31, A*11, A*23, and A*29 (20% each) (Fig 2A and Table 1). All eight tetramer-negative patients showed a heterozygous pattern, with the most common allele being A*24 (75% of the donors), followed by A*31 (37.5%) and A*68 (37.5%) (Table 1). Two individuals of the HD group had HLA-A24 homozygous alleles, and A*24 was the most common allele (57.0% of the donors) observed in a similar proportion of the chagasic patients (Table 1).

To determine the relationship between the HLA allele supertype and TcTLE recognition, tetramer- (twenty) or cytokine/cytotoxic-positive (eight) patients and healthy controls (twelve) were subjected to medium-high-resolution molecular typing (Table 2). Seven HLA-A homozygous patients were identified: five A*2402, one A*2301 and one A*0101. Note that the A*2402 and A*2301 alleles belong to the A24 supertype and that the A*0101 allele belongs to the A1 supertype (Fig 2B and Table 2). Additionally, 14 HLA-A heterozygous patients were found to have the A*2402 allele (57.1%) in the absence of A*6802 (Fig 2B), which belongs to the A2 supertype, the same supertype of the A*0201 allele in which the TcTLE peptide presentation was first described. Indeed, the A*2402 allele was the most frequent allele in the A*24 individuals (94.1%), (Fig 2B and Table 2). Additionally, two A1 supertype-allele members were detected in chagasic patients: one patient had A*3002/A*3201 and two patients had A*0101 alleles, in the absence of any A2 or A24 supertype members (Fig 2B and Table 2). In contrast, six of the eight A*68 patients were A*6802.

**Table 2:** Medium-high-resolution HLA typing and supertype groups.

Patient code	HLA-A (SSP Unitray)	Types of HLA-A alleles	Supertype group	Patient code	HLA-A (SSP Unitray)	Types of HLA-A alleles	Supertype group
**KT-035**	A*23	A*2301	A24	**KT-058**	A*24	A*2402	A24
	A*24	A*2402	A24		A*68	A*6802	A2
**KT-094**	A*24	A*2403	A24	**KT-061**	A*24	A*2402	A24
	A*68	A*6801	A3		A*31	A*3101	A3
**KT-096**	A*01	A*0101	A1	**KT-063**	A*11	A*1101	A3
	A*24	A*2402	A24		A*68	A*6802	A2
**KT-097**	A*24	A*2414	A24	**KT-067**	A*01	A*0101	A1
	A*24	A*2414	A24		A*31	A*3109	A3
**KT-098**	A*30	A*3002	A1	**KT-072**	A*23	A*2301	A24
	A*33	A*3301	A3		A*23	A*2301	A24
**KT-099**	A*03	A*0301	A3	**KT-074**	A*11	A*1101	A3
	A*68	A*6801	A3		A*24	A*2402	A24
**KT-100**	A*24	A*2404	Unclassified	**KT-078**	A*01	A*0101	A1
	A*30	A*3001	A1/A3		A*29	A*2904	Unclassified
**KT-101**	A*24	A*2402	A24	**KT-087**	A*24	A*2402	A24
	A*30	A*3001	A1/A3		A*68	A*6801	A3
**CS-005**	A*29	A*2903	A1/A24	**KT-088**	A*24	A*2402	A24
	A*31	A*3101	A3		A*24	A*2402	A24
**CS-007**	A*24	A*2402	A24	**QX-002**	A*24	A*2402	A24
	A*24	A*2402	A24		A*31	A*3101	A3
**CS-016**	A*24	A*2402	A24	**QX-018**	A*24	A*2402	A24
	A*68	A*6801	A3		A*24	A*2402	A24
**CS-017**	A*11	A*1101	A3	**QX-031**	A*24	A*2402	A24
	A*33	A*3301	A3		A*24	A*2402	A24
**CS-018**	A*30	A*3001	A1/A3	**QX-035**	A*24	A*2402	A24
	A*33	A*3301	A3		A*30	A*3010	Unclassified
**CS-040**	A*03	A*0301	A3	**QX-036**	A*24	A*2402	A24
	A*30	A*3004	A1		A*68	A*6801	A3
**KT-034**	A*23	A*2301	A24	**QX-039**	A*24	A*2402	A24
	A*68	A*6802	A2		A*29	A*2902	A1/A24
**KT-039**	A*24	A*2402	A24	**QX-045**	A*01	A*0101	A1
	A*24	A*2402	A24		A*01	A*0101	A1
**KT-040**	A*30	A*3002	A1	**QX-050**	A*03	A*0301	A3
	A*32	A*3201	A1		A*30	A*3001	A1/A3
**KT-043**	A*23	A*2301	A24	**QX-051**	A*11	A*1101	A3
	A*68	A*6802	A2		A*24	A*2402	A24
**KT-044**	A*24	A*2402	A24	**QX-052**	A*29	A*2902	A1/A24
	A*24	A*2402	A24		A*31	A*3101	A3
**KT-051**	A*11	A*1101	A3	**QX-054**	A*24	A*2402	A24
	A*23	A*2301	A24		A*68	A*6802	A2
**KT-053**	A*24	A*2414	A24	**QX-062**	A*24	A*2402	A24
	A*68	A*6802	A2		A*29	A*2902	A1/A24

### Functional activity of TcTLE peptide-specific CD8^+^ T cells in HLA-A2-negative (A24 and A1 supertypes) chagasic patients

To evaluate the functional activity of TcTLE peptide-specific CD8^+^ T cells from HLA-A2-negative patients with A24 or A1 supertypes, CD8^+^ T cells from twelve chronic chagasic patients were analyzed using six functional parameters after TcTLE peptide stimulation. Thus, the intracellular production of three cytokines (IFN-γ, TNFα and IL-2) and the potential cytotoxic activity, which was measured by evaluating CD107a/b expression as a marker of degranulation and intracellular perforin and granzyme B expression, were assessed.

**Fig 3:**
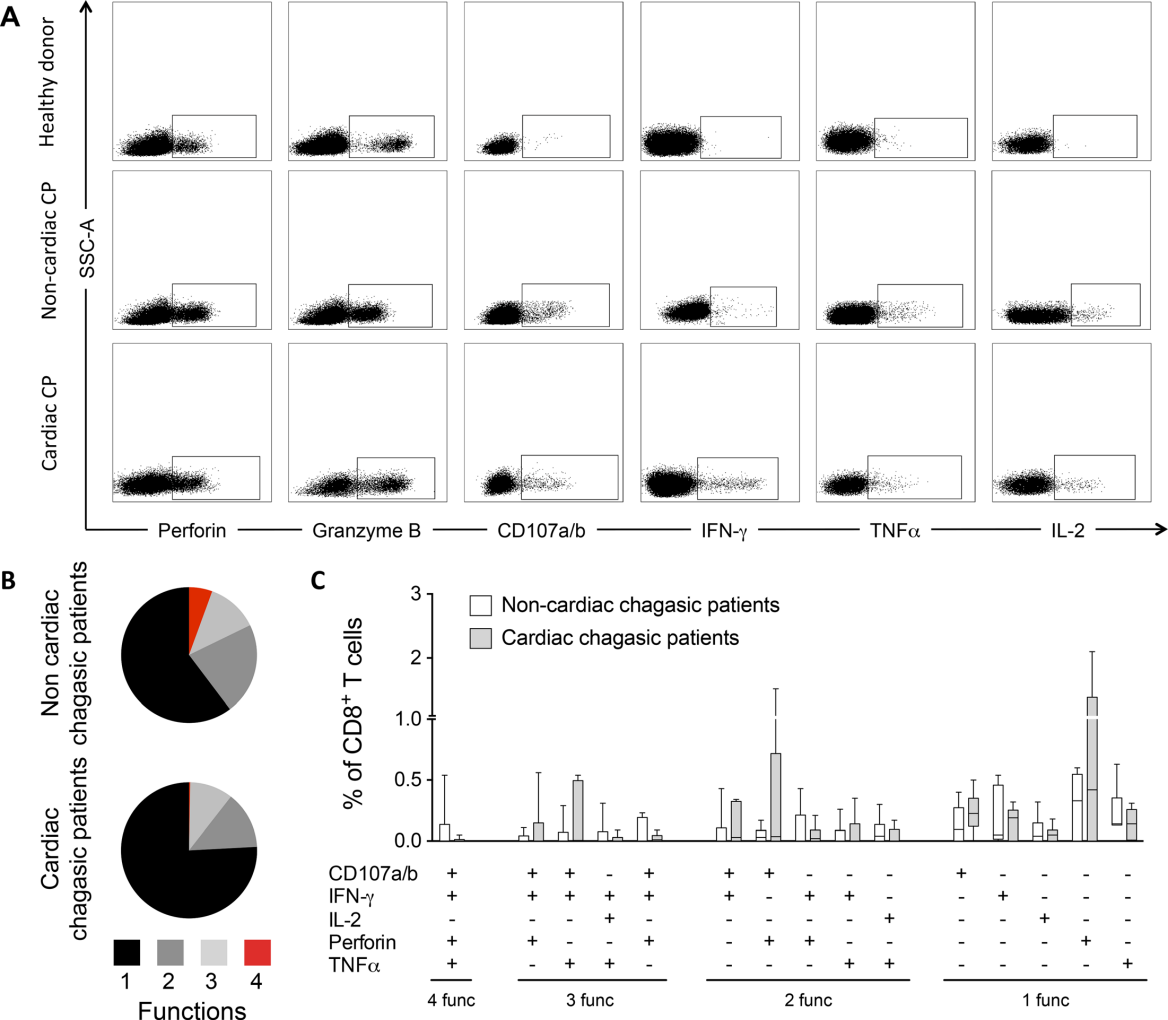
Polyfunctional profile of TcTLE-specific CD8^+^ T cell responses. (A) Functional cellular analysis of parasite-specific CD8^+^ T cells after stimulation with the TcTLE peptide. Gates were applied to identify cytokine-positive cells; these were defined according to the unstimulated samples for each subject. (B) The functional profiles of CD8^+^ T cells of HLA-A2-negative chagasic chronic patients were determined using a five-function detection method for CD107a/b, perforin, IFN-γ, IL-2 and TNFα after stimulation with the TcTLE peptide. The pie charts show the median percentages of responding CD8^+^ T cells grouped according to the number of simultaneous functions performed and color-coded depending on the functional profile. (C) Frequency of TcTLE-specific CD8^+^ T cells producing fifteen distinct combinations of five functions in non-CCC (white bars) and CCC patients (black bars). The results are shown as box-and-whisker (min-to-max) plots with median percentages of responding CD8^+^ T cells.

The flow cytometric analysis strategy is shown in Fig 3A. Analysis of individual cytokine production showed that all chagasic patients had CD8^+^ T cells that produced at least one of these cytokines (Table 3). As shown, one of twelve (8.3%) patients showed CD8^+^ T cells with only one or two effector functions, two patients (16.6%) showed three functions, three patients (25%) exhibited four functions, and four patients (33.3%) presented positive results for five of the six molecules tested. Cytokine-producing CD8^+^ T cells were not detected in HDs after antigenic stimulation (Table 3).

**Table 3:** Frequency of cytokine response and cytotoxic activity after TcTLE peptide stimulation.

Patient code	Genotype	Clinical status	Frequency of cytokine production after TcTLE peptide stimulation ^a^					
			IFN-γ	TNFα	IL-2	Perforin	Granzyme B	CD107a/b
CS-005	A*2903/A*3101	HD	0	0.03	0.00	0.00	0.00	0.00
CS-007	A*2402/A*2402	HD	0.01	0.00	0.03	0.00	0.00	0.04
CS-016	A*2402/A*6801	HD	0.04	0.00	0.00	0.00	0.00	0.00
CS-017	A*1101/A*3301	HD	0.01	0.00	0.00	0.00	0.00	0.00
CS-018	A*3001/A*3301	HD	0.01	0.00	0.00	0.00	0.00	0.00
CS-040	A*0301/A*3004	HD	0.02	0.01	0.00	0.00	0.03	0.04
QX-031	A*2402/A*2402	G0	**0.57**	0.00	0.03	0.00	0.00	0.00
QX-035	A*2402/A*3010	G0	0.03	**0.90**	0.00	**3.70**	**2.40**	**0.07**
QX-036	A*2402/A*6801	G0	0.00	**0.07**	0.01	0.00	**1.10**	**0.60**
QX-039	A*2402/A*2902	G0	**0.47**	**0.15**	0.02	**8.60**	**2.30**	**0.22**
QX-045	A*0101/A*0101	G0	**0.09**	**0.19**	**0.09**	**2.50**	**0.80**	0.00
QX-050	A*0301/A*3001	G0	**0.36**	0.00	**0.11**	**2.45**	0.00	0.00
QX-052	A*2902/A*3101	G2	**0.62**	**0.56**	**0.07**	**3.60**	**0.07**	**0.76**
QX-002	A*2402/A*3101	G3	**0.10**	0.00	**0.09**	**2.37**	**5.70**	**0.41**
QX-031	A*2402/A*2402	G3	**0.45**	**0.52**	**0.40**	0.00	0.00	**0.18**
QX-051	A*1101/A*2402	G3	**0.59**	**0.09**	**0.32**	0.00	**0.10**	0.00
QX-054	A*2402/A*6802	G3	0.00	**0.33**	0.00	0.03	**4.80**	0.00
QX-062	A*2402/A*2902	G3	**0.49**	0.00	**0.10**	**1.29**	**2.60**	**0.50**

^a^ A positive cytokine response (bold) was defined as > 0.05% after background (without antigen) subtraction.

Subsequently, CD8^+^ T cell polyfunctionality, which refers to simultaneous cytokine production and cytotoxic activity by a single cell, was evaluated. Five effector functions (IFN-γ, TNFα, IL-2, perforin and CD107a/b) were measured after peptide stimulation. No TcTLE-specific CD8^+^ T cells exhibited all five effector functions (Fig 3B and 3C) in non-cardiac or CCC patients. In contrast, the peptide-specific CD8^+^ T cells of CCC and non-CCC patients presented positive results for four, three and two of these functions (Fig 3C). Generally, non-CCC patients had a higher frequency of CD8^+^ T cells exhibiting four or two functions than those with cardiac manifestations. Thus, the most predominant profile including four positive effector functions was CD107a/b, IFN-γ, perforin and TNFα. In this case, although no significant differences were found, non-CCC patients tended to have more polyfunctional CD8^+^ T cells than did CCC patients. In contrast, specific CD8^+^ T cells with three positive functions were observed at similar frequencies in both patient groups. Although significant differences were not observed, the expression of IFN-γ and perforin was more frequent in non-CCC patients, whereas the production of CD107a/b and perforin was more frequent in CCC patients. Regarding monofunctional cells, TNFα and IFN-γ were the major cytokines produced by CD8^+^ T cells from non-CCC patients, and perforin was the most predominant functionality detected in CD8^+^ T cells from CCC patients (Fig 3C). Overall, these results demonstrate that TcTLE peptide-specific CD8^+^ T cells from HLA-A2 negative chagasic patients (specifically A24 and A1 supertypes) are functional.

## Discussion

Using experimental animal models, CD8^+^ T cells were demonstrated to be essential in controlling the spread of *T*. *cruzi* infection [3–8]. In addition, CD8^+^ T cells specific to *T*. *cruzi* antigens play an important role in protecting against *T*. *cruzi* infection in humans, and most of their epitopes are restricted to the HLA-A*0201 allele. Thus, some A2-restricted epitopes have been extensively studied to explore their use in vaccine or immunotherapy design [29–32]. One of these epitopes, the TcTLE peptide, has been reported to be a promiscuous epitope presented in several alleles from the A2 supertype [22, 27]. In the in-depth study described here, the TcTLE peptide was also presented by other HLA-A alleles belonging to the A1 and A24 supertypes. Consequently, the functional activity of the TcTLE-specific CD8^+^ T cells from these HLA-A2-negative chronic chagasic patients was analyzed in terms of frequency and functionality, and its correlation with patient haplotype was assessed.

Several reports have described promiscuous T cell epitopes derived from infectious organisms, such as the dengue and Epstein-Barr (EBV) viruses, which are presented by more than one HLA-A allele and even by different HLA-A supertypes [17,18]. For example, *Mongkolsapaya et al*. [18] reported the promiscuous presentation of an A*2402-restricted epitope (from the A24 supertype) and an A*1101 allele, classified in the A3 supertype. Additionally, *Liu et al*. [16] described various influenza A virus peptides capable of being presented by alleles belonging to the A24 and A3 supertypes. In this study, the promiscuous recognition of the TcTLE peptide among CD8^+^ T cells from chagasic donors having A1, A2 and A24 supertypes was detected. This phenomenon of presentation by different MHC supertypes has also been identified in tumor-derived peptides [20,33].

Peptides from different pathogens presented in the context of homozygous HLA-A alleles confirmed the antigen presentation by alleles belonging to different supertypes. In *Mycobacterium tuberculosis*, peptides derived from the TB10.4 protein are presented by homozygous HLA-A*3001 and A*3002 alleles, which are recognized by peptide-specific CD8^+^ T cells from patients with active tuberculosis [34]. Cytomegalovirus AYAQKIFKIL- and PYLFWLAAI EBV-derived-peptides are presented by both HLA A*2301 and A*2402 alleles and generate a CTL response [35]. Similarly, the TcTLE peptide was presented by different homozygous HLA-A alleles, such as A*0101 from the A1 supertype and A*2301 and A*2402 from the A24 supertype.

Although HLA-B and HLA-C alleles may also bind and present the TcTLE peptide, our results indicate that the TcTLE peptide was able to be presented by the HLA-A*6802 allele from the A2 supertype, the supertype in which this peptide was originally described, and by other HLA-A alleles belonging to other supertypes, such as HLA-A*0101, A*3002, A*3201 and A*1101 of the A1 supertype) and A*2301 and A*2402 of the A24 supertype, which all generated measurable specific CD8^+^ T cell responses in *T*. *cruzi*-infected patients.

Using tetramer staining, CD8^+^ T cells from fifteen of nineteen (78.9%) HLA-A2-positive chagasic patients were previously shown to recognize the TcTLE peptide with tetramer frequencies ranging from 0.09% to 0.34% [22]. Similar percentages and frequencies are reported here: twenty of twenty-eight (71.4%) HLA-A2-negative chagasic patients had tetramer frequencies of 0.07‒0.37%. Consequently, antigen-specific CD8^+^ T cells from chronic chagasic patients recognized the TcTLE peptide independent of their HLA-A supertype, which indirectly indicates that the TcTLE peptide is presented by HLA-A alleles other than HLA-A2. Taken into account the published data [12,36–41] summarized in Table 4, the binding of the TcTLE (T**L**EEFSAK**L**) peptide to other alleles of the HLA-A1 and HLA-A24 supertypes is possible because the leucine (L) at 2 and 9 primary anchors positions of the TcTLE peptide bound to the B and F pockets of the MHC molecules of these supertypes (Table 4). Regarding the Flu-MP* peptide, previously we described that it is recognized by several alleles from the A2 supertype. The results of this work, suggest that this peptide could be also presented by other alleles from different supertypes. This possibility can be explained by the fact that the isoleucine (I) at 2 position and the leucine (L) at 9 position of the Flu-MP* (G**I**LGFVTT**L**) peptide, fits in the B and F pockets of the alleles of these supertypes, respectively (Table 4). However, this hypothesis should be confirmed.

**Table 4:** Summary of primary anchors influences on the HLA-A2, A1 and A24 supertypes binding affinity.

Supertype	Allele(s)	Position 2 motif	Position 9 motif	References
A2	A*0201	LMV [TQAI]	VIL [MTA]	[12,37–41]
	A*6802			
A1	A*0101	TI [LMVS]	FWY [LV]	[36,38–40]
	A*3201			
A24	A*2402	YF [LMVIT]	FI [WLMY]	[36,38–40]
	A*2301			

Amino acids listed in brackets are residues that are tolerated.

The analysis of individual cytokine production showed that all HLA-A2-negative chagasic patients had CD8^+^ T cells that produced at least one of the cytokines tested. This finding, together with the existence of polyfunctional CD8^+^ T cells in these HLA-A2-negative chagasic patients, allow us to propose that these CD8^+^ T cells from chronic chagasic patients belonging to the A24 and A1 supertypes are fully functional. Regarding the heterogeneous phenotype of the CD8^+^ T cells exhibiting the antagonistic functional activities associated with protection or pathogenesis, for instance, IFN-γ versus perforin [3], a larger sample size is needed to reach a conclusion concerning the role of heterogeneous phenotypes in disease control or progression. A similar requirement is suggested for assessing the role of the polyfunctional CD8^+^ T cells, which are usually associated with protection against chronic infections [42,43]. Indeed, recent results from our group indicate that patients with less-severe disease have a higher frequency of polyfunctional CD8^+^ T cells, whereas patients at the advanced stage of the disease have a higher frequency of monofunctional CD8^+^ T cells [44]. The fact that the total parasite antigens, the KMP-11 protein [44] and the TcTLE peptide have revealed that the disease severity in cardiac chronic chagasic patients could be associated with a low frequency of polyfunctional CD8^+^ T cells, suggest that these markers could be evaluated for their use in the prediction of progression disease in accordance with the polyfunctional potential loss.

The promiscuity of the TcTLE peptide was observed both by soluble tetramers assays and cytokine production after peptide stimulation. Therefore, the cytokine production and the cytotoxic activity results reinforce the findings of TcTLE peptide promiscuity, according to the results found with tetramers. In addition, the frequency of TcTLE peptide-specific non-CD8^+^ T cells was evaluated and no frequencies of non-CD8^+^ T cells specific to this peptide were found (S1 Fig), finding that the tetramer binding is specific. Taking into account that subjects with ongoing chronic inflammation/immune stimulation due to chronic infections or inflammatory diseases are likely to have higher background staining with tetramer reagents, it is important to mention that we previously reported that patients with non-chagasic chronic heart diseases did not have significant frequencies of TcTLE peptide-specific CD8^+^ T cells [45]. However, other approaches as the use APCs consisting of HLA empty cell lines expressing only the HLA allele of interest could be used to further explore the specificity of HLA epitope restriction.

The most common HLA-A alleles in the Latin American population are as follows: HLA- A*02 (28%), A*24 (11%) and A*68 (5%) in Brazil [46,47]; A*02 (50%), A*24 (14%) and A*68 (10%) in Bolivia [48]; A*02 (63.4%), A*30 (10.2%), A*24 (6.6%) and A*68 (5.4%) in Peru [49]; and HLA-A*02 (25.5%), A*24 (23%) and A*68 (6.0%) in Colombia [50,51]. Notably, these reports revealed that more than 45% of the Latin American population have HLA-A*02, A*24 or A*68 HLA alleles; A*02 was the original allele restriction reported for the TcTLE peptide [21,22]. In this work, A*24 and A*68 alleles were also implicated in TcTLE presentation.

Consequently, a high percentage of the Latin American population has HLA-A alleles belonging to the A1, A2 and A24 supertypes, which are capable of presenting the TcTLE peptide and of inducing immune responses through effector functions and cytotoxic activities. Additionally, the TcTLE sequence is conserved among different parasite strains, particularly if it is also present in the KMP-11 protein from *Trypanosoma rangeli*, a synanthropic non-human pathogenic parasite [52]. Taken together, these results reasonably suggest that the TcTLE peptide, together with other parasite sequences [53,54], could represent a potential target for vaccine or immunotherapy development against Chagas disease.

In summary, these results demonstrate that the TcTLE peptide epitope is recognized and presented by different HLA-A alleles from the A2, A24 and A1 supertypes and that it induces an immune response characterized by cytokine production and cytotoxic activity. This *T*. *cruzi*-derived peptide, which exhibits broad coverage of HLA presentation in the population, is a potential target for immunotherapy against Chagas disease.

## Supporting Information

S1 Fig Frequency of TcTLE-specific CD8^+^ T cells and non-CD8^+^ T cells. Dot plots representative of the analysis of two HLA-A2-negative chagasic patients. Analysis were made on total CD3^+^ T cells to evaluated the frequency of TcTLE-specific CD8^+^ T cells and non-CD8^+^ T cells, and on total CD8^+^ T cells.
(TIF)

S1 Table Frequency of cytokine response and cytotoxic activity after culture without stimulation. (PDF)

S2 Table Frequency of cytokine response and cytotoxic activity after SEB (positive control) stimulation. (PDF)

## References

[1] WHO. Chagas disease (American trypanosomiasis)—fact sheet (revised in August 2012). Wkly Epidemiol Rec. 2012; 87: 519–522. 23311009

[2] WHO. Chagas disease in Latin America: an epidemiological update based on 2010 estimates. Wkly Epidemiol Rec. 2015; 90: 33–43. 25671846

[3] Silverio JC, Pereira IR, Cipitelli Mda C, Vinagre NF, Rodrigues MM, Gazzinelli RT, et al CD8+ T-cells expressing interferon gamma or perforin play antagonistic roles in heart injury in experimental *Trypanosoma cruzi*-elicited cardiomyopathy. PLoS Pathog. 2012; 8: e1002645 10.1371/journal.ppat.1002645 22532799 PMC3330123

[4] Martin D, Tarleton R. Generation, specificity, and function of CD8^+^ T cells in *Trypanosoma cruzi* infection. Immunol Rev. 2004; 201: 304–317. 15361249 10.1111/j.0105-2896.2004.00183.x

[5] Rottenberg ME, Bakhiet M, Olsson T, Kristensson K, Mak T, Wigzell H, et al Differential susceptibilities of mice genomically deleted of CD4^+^ and CD8^+^ to infections with *Trypanosoma cruzi* or *Trypanosoma brucei*. Infect Immun. 1993; 61: 5129–5133. 8225589 10.1128/iai.61.12.5129-5133.1993 PMC281292

[6] Tarleton RL. Depletion of CD8^+^ T cells increases susceptibility and reverses vaccine-induced immunity in mice infected with *Trypanosoma cruzi*. J Immunol. 1990; 144: 717–724. 2104903

[7] Tarleton RL, Koller BH, Latour A, Postan M. Susceptibility of beta 2-microglobulin-deficient mice to *Trypanosoma cruzi* infection. Nature. 1992; 356: 338–340. 1549177 10.1038/356338a0

[8] Tarleton RL, Sun J, Zhang L, Postan M. Depletion of T-cell subpopulations results in exacerbation of myocarditis and parasitism in experimental Chagas' disease. Infect Immun. 1994; 62: 1820–1829. 8168945 10.1128/iai.62.5.1820-1829.1994 PMC186416

[9] A. BF, C. OH. Adaptive Immunity 2010.

[10] Grommé M, Neefjes J. Antigen degradation or presentation by MHC class I molecules via classical and non-classical pathways. Mol Immunol. 2002; 39: 181–202. 12200050 10.1016/s0161-5890(02)00101-3

[11] Yewdell JW, Reits E, Neefjes J. Making sense of mass destruction: quantitating MHC class I antigen presentation. Nat Rev Immunol. 2003; 3: 952–961. 14647477 10.1038/nri1250

[12] Doytchinova I, Flower D. The HLA-A2-supermotif: a QSAR definition. Org Biomol Chem. 2003; 1: 2648–2654. 12948188 10.1039/b300707c

[13] Sidney J, Peters B, Frahm N, Brander C, Sette A. HLA class I supertypes: a revised and updated classification. BMC Immunol. 2008; 9: 1 10.1186/1471-2172-9-1 18211710 PMC2245908

[14] Hertz T, Yanover C. Identifying HLA supertypes by learning distance functions. Bioinformatics. 2007; 23: e148–155. 17237084 10.1093/Bioinformatics/btl324

[15] Thomsen M, Lundegaard C, Buus S, Lund O, Nielsen M. MHCcluster, a method for functional clustering of MHC molecules. Immunogenetics. 2013; 65: 655–665. 10.1007/s00251-013-0714-9 23775223 PMC3750724

[16] Liu J, Zhang S, Tan S, Yi Y, Wu B, Cao B, et al Cross-allele cytotoxic T lymphocyte responses against 2009 pandemic H1N1 influenza A virus among HLA-A24 and HLA-A3 supertype-positive individuals. J Virol. 2012; 86: 13281–13294. 10.1128/JVI.01841-12 23015716 PMC3503122

[17] Frahm N, Yusim K, Suscovich TJ, Adams S, Sidney J, Hraber P, et al Extensive HLA class I allele promiscuity among viral CTL epitopes. Eur J Immunol. 2007; 37: 2419–2433. 17705138 10.1002/eji.200737365 PMC2628559

[18] Mongkolsapaya J, Duangchinda T, Dejnirattisai W, Vasanawathana S, Avirutnan P, Jairungsri A, et al T cell responses in dengue hemorrhagic fever: are cross-reactive T cells suboptimal? J Immunol. 2006; 176: 3821–3829. 16517753 10.4049/jimmunol.176.6.3821

[19] Sidney J, Grey HM, Southwood S, Celis E, Wentworth PA, del Guercio MF, et al Definition of an HLA-A3-like supermotif demonstrates the overlapping peptide-binding repertoires of common HLA molecules. Hum Immunol. 1996; 45: 79–93. 8882405 10.1016/0198-8859(95)00173-5

[20] Terasaki Y, Shichijo S, Niu Y, Komatsu N, Noguchi M, Todo S, et al An HLA-A3-binding prostate acid phosphatase-derived peptide can induce CTLs restricted to HLA-A2 and -A24 alleles. Cancer Immunol Immunother. 2009; 58: 1877–1885. 10.1007/s00262-009-0699-2 19330328 PMC11030184

[21] Maranon C, Thomas MC, Planelles L, Lopez MC. The immunization of A2/K(b) transgenic mice with the KMP11-HSP70 fusion protein induces CTL response against human cells expressing the *T*. *cruzi* KMP11 antigen: identification of A2-restricted epitopes. Mol Immunol. 2001; 38: 279–287. 11566321 10.1016/s0161-5890(01)00059-1

[22] Lasso P, Mesa D, Cuellar A, Guzman F, Bolanos N, Rosas F, et al Frequency of specific CD8^+^ T cells for a promiscuous epitope derived from *Trypanosoma cruzi* KMP-11 protein in chagasic patients. Parasite Immunol. 2010; 32: 494–502. 10.1111/j.1365-3024.2010.01206.x 20591120

[23] Beltrán M, Duque S, Guhl F, Herrera CP, López MC, Moreno AL, et al (2001) Prueba de ELISA y prueba de inmunofluorescencia indirecta (IFI) In: Guhl F, Nicholls RS, editors. Manual de procedimientos para el diagnóstico de la enfermedad de Chagas. Bogotá: Ministerio de Salud pp. 32–48.

[24] Kuschnir E, Sgammini H, Castro R, Evequoz C, Ledesma R, Brunetto J. [Evaluation of cardiac function by radioisotopic angiography, in patients with chronic Chagas cardiopathy]. Arq Bras Cardiol. 1985; 45: 249–256. 3835868

[25] Parham P, Brodsky FM. Partial purification and some properties of BB7.2. A cytotoxic monoclonal antibody with specificity for HLA-A2 and a variant of HLA-A28. Hum Immunol. 1981; 3: 277–299. 7035415 10.1016/0198-8859(81)90065-3

[26] Lasso P, Mesa D, Bolanos N, Cuellar A, Guzman F, Cucunuba Z, et al Chagasic patients are able to respond against a viral antigen from influenza virus. BMC Infect Dis. 2012; 12: 198 10.1186/1471-2334-12-198 22920436 PMC3511223

[27] Roederer M, Nozzi JL, Nason MC. SPICE: exploration and analysis of post-cytometric complex multivariate datasets. Cytometry A. 2011; 79: 167–174. 10.1002/cyto.a.21015 21265010 PMC3072288

[28] Diez H, López MC, Del Carmen Thomas M, Guzmán F, Rosas F, Velazco V, et al Evaluation of IFN-gamma production by CD8^+^ T lymphocytes in response to the K1 peptide from KMP-11 protein in patients infected with *Trypanosoma cruzi*. Parasite Immunol. 2006; 28: 101–105. 16441508 10.1111/j.1365-3024.2005.00815.x

[29] Egui A, Thomas MC, Morell M, Maranon C, Carrilero B, Segovia M, et al *Trypanosoma cruzi* paraflagellar rod proteins 2 and 3 contain immunodominant CD8^+^ T-cell epitopes that are recognized by cytotoxic T cells from Chagas disease patients. Mol Immunol. 2012; 52: 289–298. 10.1016/j.molimm.2012.05.021 22750229

[30] Fonseca SG, Moins-Teisserenc H, Clave E, Ianni B, Nunes VL, Mady C, et al Identification of multiple HLA-A*0201-restricted cruzipain and FL-160 CD8^+^ epitopes recognized by T cells from chronically *Trypanosoma cruzi*-infected patients. Microbes Infect. 2005; 7: 688–697. 15848276 10.1016/j.micinf.2005.01.001

[31] Marañón C, Egui A, Carrilero B, Thomas MC, Pinazo MJ, Gascón J, et al Identification of HLA-A*02:01-restricted CTL epitopes in *Trypanosoma cruzi* heat shock protein-70 recognized by Chagas disease patients. Microbes Infect. 2011; 13: 1025–1032. 10.1016/j.micinf.2011.05.010 21704723

[32] Martin DL, Weatherly DB, Laucella SA, Cabinian MA, Crim MT, Sullivan S, et al CD8^+^ T-Cell responses to *Trypanosoma cruzi* are highly focused on strain-variant trans-sialidase epitopes. PLoS Pathog. 2006; 2: e77 16879036 10.1371/journal.ppat.0020077 PMC1526708

[33] Mohamed ER, Naito M, Terasaki Y, Niu Y, Gohara S, Komatsu N, et al Capability of SART3(109–118) peptide to induce cytotoxic T lymphocytes from prostate cancer patients with HLA class I-A11, -A31 and -A33 alleles. Int J Oncol. 2009; 34: 529–536. 19148489

[34] Axelsson-Robertson R, Ahmed RK, Weichold FF, Ehlers MM, Kock MM, Sizemore D, et al Human leukocyte antigens A*3001 and A*3002 show distinct peptide-binding patterns of the *Mycobacterium tuberculosis* protein TB10.4: consequences for immune recognition. Clin Vaccine Immunol. 2011; 18: 125–134. 10.1128/CVI.00302-10 21084459 PMC3019778

[35] Burrows SR, Elkington RA, Miles JJ, Green KJ, Walker S, Haryana SM, et al Promiscuous CTL recognition of viral epitopes on multiple human leukocyte antigens: biological validation of the proposed HLA A24 supertype. J Immunol. 2003; 171: 1407–1412. 12874232 10.4049/jimmunol.171.3.1407

[36] Vita R, Overton JA, Greenbaum JA, Ponomarenko J, Clark JD, Cantrell JR, et al The immune epitope database (IEDB) 3.0. Nucleic Acids Res. 2015; 43: D405–412. 10.1093/nar/gku938 25300482 PMC4384014

[37] Sidney J, Southwood S, Mann DL, Fernandez-Vina MA, Newman MJ, Sette A. Majority of peptides binding HLA-A*0201 with high affinity crossreact with other A2-supertype molecules. Hum Immunol. 2001; 62: 1200–1216. 11704282 10.1016/s0198-8859(01)00319-6

[38] Sette A, Sidney J. Nine major HLA class I supertypes account for the vast preponderance of HLA-A and -B polymorphism. Immunogenetics. 1999; 50: 201–212. 10602880 10.1007/s002510050594

[39] Lund O, Nielsen M, Kesmir C, Petersen AG, Lundegaard C, Worning P, et al Definition of supertypes for HLA molecules using clustering of specificity matrices. Immunogenetics. 2004; 55: 797–810. 14963618 10.1007/s00251-004-0647-4

[40] Kubo RT, Sette A, Grey HM, Appella E, Sakaguchi K, Zhu NZ, et al Definition of specific peptide motifs for four major HLA-A alleles. J Immunol. 1994; 152: 3913–3924. 8144960

[41] Falk K, Rotzschke O, Stevanovic S, Jung G, Rammensee HG. Allele-specific motifs revealed by sequencing of self-peptides eluted from MHC molecules. Nature. 1991; 351: 290–296. 1709722 10.1038/351290a0

[42] Darrah PA, Patel DT, De Luca PM, Lindsay RW, Davey DF, Flynn BJ, et al Multifunctional TH1 cells define a correlate of vaccine-mediated protection against *Leishmania major*. Nat Med. 2007; 13: 843–850. 17558415 10.1038/nm1592

[43] Forbes EK, Sander C, Ronan EO, McShane H, Hill AV, Beverley PC, et al Multifunctional, high-level cytokine-producing Th1 cells in the lung, but not spleen, correlate with protection against *Mycobacterium tuberculosis* aerosol challenge in mice. J Immunol. 2008; 181: 4955–4964. 18802099 10.4049/jimmunol.181.7.4955 PMC2867031

[44] Lasso P, Mateus J, Pavia P, Rosas F, Roa N, Thomas MC, et al Inhibitory Receptor Expression on CD8+ T Cells Is Linked to Functional Responses against Trypanosoma cruzi Antigens in Chronic Chagasic Patients. J Immunol. 2015; 195: 3748–3758. 10.4049/jimmunol.1500459 26385520

[45] Giraldo NA, Bolanos NI, Cuellar A, Guzman F, Uribe AM, Bedoya A, et al Increased CD4^+^/CD8^+^ double-positive T cells in chronic Chagasic patients. PLoS Negl Trop Dis. 2011; 5: e1294 10.1371/journal.pntd.0001294 21886854 PMC3160296

[46] Bardi MS, Jarduli LR, Jorge AJ, Camargo RB, Carneiro FP, Gelinski JR, et al HLA-A, B and DRB1 allele and haplotype frequencies in volunteer bone marrow donors from the north of Parana State. Rev Bras Hematol Hemoter. 2012; 34: 25–30. 10.5581/1516-8484.20120010 23049380 PMC3459602

[47] Bortolotto AS, Petry MG, da Silveira JG, Raya AR, Fernandes SR, Neumann J, et al HLA-A, -B, and -DRB1 allelic and haplotypic diversity in a sample of bone marrow volunteer donors from Rio Grande do Sul State, Brazil. Hum Immunol. 2012; 73: 180–185. 10.1016/j.humimm.2011.11.009 22154725

[48] Martinez-Laso J, Siles N, Moscoso J, Zamora J, Serrano-Vela JI, R-A-Cachafeiro JI, et al Origin of Bolivian Quechua Amerindians: their relationship with other American Indians and Asians according to HLA genes. Eur J Med Genet. 2006; 49: 169–185. 16530714 10.1016/j.ejmg.2005.04.005

[49] Moscoso J, Seclen S, Serrano-Vela JI, Villena A, Martinez-Laso J, Zamora J, et al HLA genes in Lamas Peruvian-Amazonian Amerindians. Mol Immunol. 2006; 43: 1881–1889. 16337001 10.1016/j.molimm.2005.10.013

[50] Arias-Murillo YR, Castro-Jiménez MA, Ríos-Espinosa MF, López-Rivera JJ, Echeverry-Coral SJ, Martínez-Nieto O. Analysis of HLA-A, HLA-B, HLA-DRB1 allelic, genotypic, and haplotypic frequencies in colombian population. Colomb Med. 2010; 41: 336–343.

[51] Arrunategui AM, Villegas A, Ocampo LA, Rodriguez LM, Badih A. Frecuencias alélicas, genotípicas y haplotípicas del sistema HLA clase I y II en donantes de una población del suroccidente colombiano. Acta Med Colomb. 2013; 38: 16–21.

[52] Diez H, Thomas MC, Urueña CP, Santander SP, Cuervo CL, López MC, et al Molecular characterization of the kinetoplastid membrane protein-11 genes from the parasite *Trypanosoma rangeli*. Parasitology. 2005; 130: 643–651. 15977901 10.1017/s0031182004006936

[53] Vasconcelos JR, Bruña-Romero O, Araújo AF, Dominguez MR, Ersching J, de Alencar BC, et al Pathogen-induced proapoptotic phenotype and high CD95 (Fas) expression accompany a suboptimal CD8+ T-cell response: reversal by adenoviral vaccine. PLoS Pathog. 2012; 8: e1002699 10.1371/journal.ppat.1002699 22615561 PMC3355083

[54] Pereira IR, Vilar-Pereira G, Marques V, da Silva AA, Caetano B, Moreira OC, et al A human type 5 adenovirus-based *Trypanosoma cruzi* therapeutic vaccine re-programs immune response and reverses chronic cardiomyopathy. PLoS Pathog. 2015; 11: e1004594 10.1371/journal.ppat.1004594 25617628 PMC4305326

