# Supplementary figures and images for: Promiscuous Recognition of a Trypanosoma cruzi CD8+ T Cell Epitope among HLA-A2, HLA-A24 and HLA-A1 Supertypes in Chagasic Patients
Source: PLoS One. 2016 Mar 14;11(3):e0150996. doi: 10.1371/journal.pone.0150996 (PMC4790940; doi:10.1371/journal.pone.0150996)

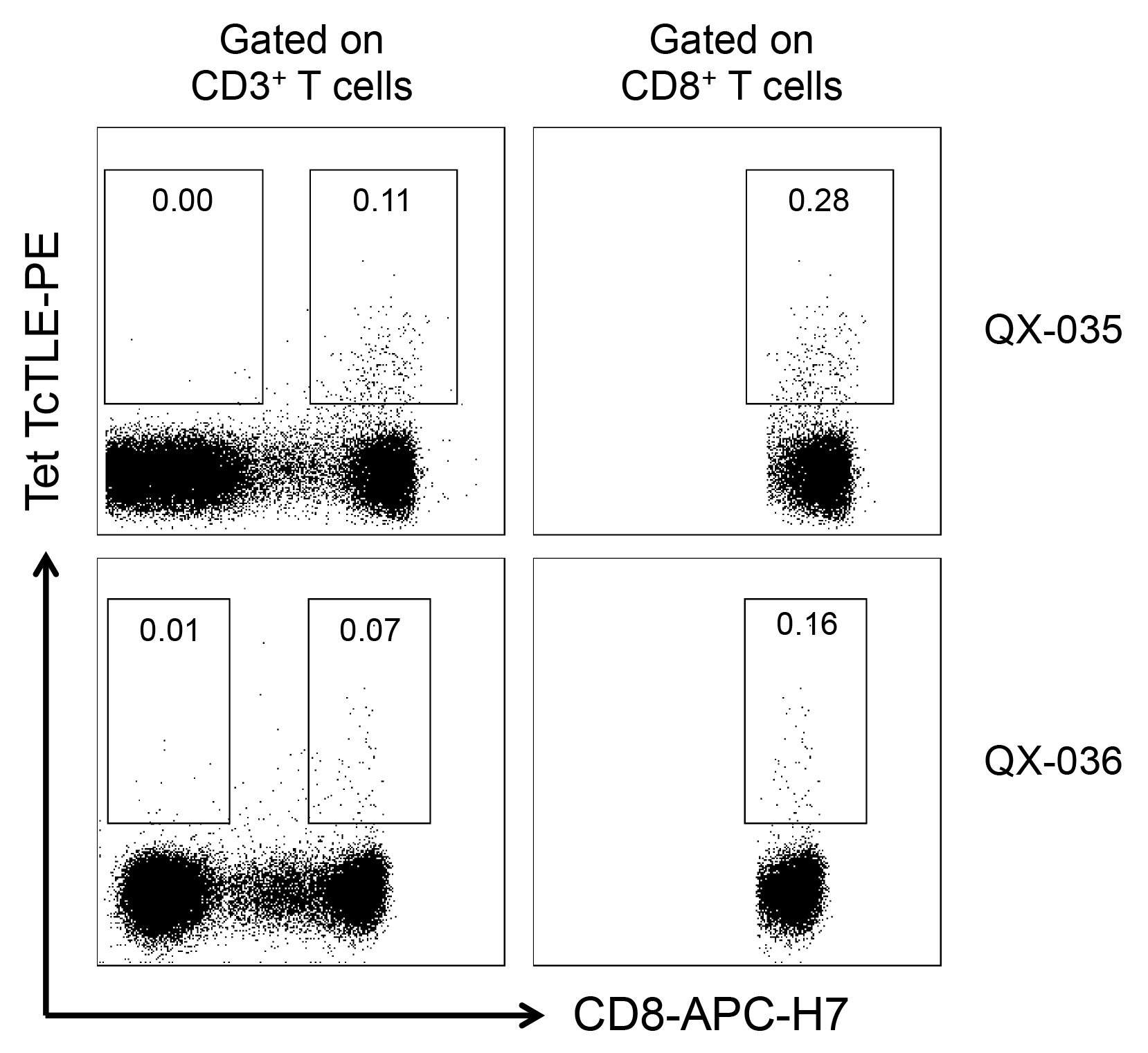

Supplement: S1 Fig — Dot plots representative of the analysis of two HLA-A2-negative chagasic patients. Analysis were made on total CD3+ T cells to evaluated the frequency of TcTLE-specific CD8+ T cells and non-CD8+ T cells, and on total CD8+ T cells. (TIF) [file pone.0150996.s001.tif]
