# Supplementary material for: Promiscuous Recognition of a Trypanosoma cruzi CD8+ T Cell Epitope among HLA-A2, HLA-A24 and HLA-A1 Supertypes in Chagasic Patients
Source: PLoS One. 2016 Mar 14;11(3):e0150996. doi: 10.1371/journal.pone.0150996 (PMC4790940; doi:10.1371/journal.pone.0150996)
Supplement: S1 Table — (PDF) [file pone.0150996.s002.pdf]

S1 Table. Frequency of cytokine response and cytotoxic activity after culture without stimulation

Patient´s code	Genotype	Clinical status	Frequency of cytokine production after TcTLE stimulation [a]					
			IFN-γ	TNFα	IL-2	Perforin	Granzyme B	CD107a/b
CS-005	A*2903/A*3101	HD	0.08	0.08	0.03	5.95	5.24	0.06
CS-007	A*2402/A*2402	HD	0.04	0.025	0.07	9.3	5.4	0.08
CS-016	A*2402/A*6801	HD	0.02	0.08	0.04	7.00	12.2	0.07
CS-017	A*1101/A*3301	HD	0.07	0.07	0.06	7.40	4.6	0.07
CS-018	A*3001/A*3301	HD	0.06	0.08	0.10	4.70	4.8	0.07
CS-040	A*0301/A*3004	HD	0.03	0.07	0.03	13.00	4.9	0.00
QX-031	A*2402/A*2402	G0	0.13	0.08	0.00	19.80	3.9	0.13
QX-035	A*2402/A*3010	G0	0.10	0.02	0.09	9.00	3.5	0.18
QX-036	A*2402/A*6801	G0	0.13	0.10	0.13	10.30	1.7	0.13
QX-039	A*2402/A*2902	G0	0.02	0.11	0.04	6.30	1.7	0.17
QX-045	A*0101/A*0101	G0	0.12	0.09	0.06	15.20	2.4	0.05
QX-050	A*0301/A*3001	G0	0.03	0.12	0.02	15.60	2.5	0.10
QX-052	A*2902/A*3101	G2	0.14	0.03	0.10	14.90	7.3	0.13
QX-002	A*2402/A*3101	G3	0.13	0.00	0.11	7.48	4.7	0.03
QX-031	A*2402/A*2402	G3	0.17	0.12	0.07	9.70	9.4	0.07
QX-051	A*1101/A*2402	G3	0.05	0.14	0.12	10.50	7.4	0.11
QX-054	A*2402/A*6802	G3	0.14	0.10	0.11	7.20	6.3	0.18
QX-062	A*2402/A*2902	G3	0.04	0.04	0.05	5.27	6.6	0.02

[a] The frequencies listed in the table are the assay background and are subtracted in the results with SEB and TcTLE stimulation.
