# Supplementary material for: Promiscuous Recognition of a Trypanosoma cruzi CD8+ T Cell Epitope among HLA-A2, HLA-A24 and HLA-A1 Supertypes in Chagasic Patients
Source: PLoS One. 2016 Mar 14;11(3):e0150996. doi: 10.1371/journal.pone.0150996 (PMC4790940; doi:10.1371/journal.pone.0150996)
Supplement: S2 Table — (PDF) [file pone.0150996.s003.pdf]

S2 Table. Frequency of cytokine response and cytotoxic activity after SEB (positive control) stimulation

Patient´s code	Genotype	Clinical status	Frequency of cytokine production after SEB stimulation [a]					
			IFN-γ	TNFα	IL-2	Perforin	Granzyme B	CD107a/b
CS-005	A*2903/A*3101	HD	2.29	2.33	1.57	1.00	0.00	1.21
CS-007	A*2402/A*2402	HD	4.30	8.09	2.63	0.00	0.00	1.87
CS-016	A*2402/A*6801	HD	5.00	3.00	3.62	0.00	1.90	5.73
CS-017	A*1101/A*3301	HD	13.13	5.19	2.19	0.00	0.00	12.13
CS-018	A*3001/A*3301	HD	11.24	5.48	5.78	0.89	0.00	10.09
CS-040	A*0301/A*3004	HD	4.50	3.99	1.12	5.60	0.00	0.92
QX-031	A*2402/A*2402	G0	3.73	3.17	1.23	4.20	4.10	2.00
QX-035	A*2402/A*3010	G0	10.05	9.54	1.61	4.80	0.50	1.67
QX-036	A*2402/A*6801	G0	14.14	13.18	7.94	1.90	0.70	10.57
QX-039	A*2402/A*2902	G0	1.52	4.70	1.79	0.40	5.50	1.15
QX-045	A*0101/A*0101	G0	13.09	11.71	3.28	2.20	0.30	13.81
QX-050	A*0301/A*3001	G0	10.57	10.69	1.92	0.00	0.00	10.73
QX-052	A*2902/A*3101	G2	20.26	17.97	2.26	7.70	0.00	16.69
QX-002	A*2402/A*3101	G3	9.83	5.00	3.67	6.32	0.00	8.13
QX-031	A*2402/A*2402	G3	10.17	10.82	1.40	0.00	0.00	8.02
QX-051	A*1101/A*2402	G3	4.16	8.00	1.12	7.20	0.00	2.43
QX-054	A*2402/A*6802	G3	18.23	19.90	11.09	3.90	10.40	12.91
QX-062	A*2402/A*2902	G3	20.76	17.75	3.57	0.10	0.00	35.57

[a] The frequencies listed in the table are the result of negative control subtraction (S1 Table).
